# MicroRNA-Transcription factor regulatory networks in the early strobilar development of *Echinococcus granulosus* protoscoleces

**DOI:** 10.1186/s12864-023-09199-3

**Published:** 2023-03-15

**Authors:** Mohammad Ali Mohammadi, Mehdi Mansouri, Ali Derakhshani, Masoud Rezaie, Mehdi Borhani, Saeid Nasibi, Seyed Mohammad Mousavi, Ali Afgar, Natalia Macchiaroli, Mara C. Rosenzvit, Majid Fasihi Harandi

**Affiliations:** 1 Research Center for Hydatid Disease in Iran, Afzalipour School of Medicine, Kerman University of Medical Sciences, Kerman, Iran; 2 Department of Agricultural Biotechnology, Faculty of Agriculture, Shahid Bahonar University of Kerman, Kerman, Iran; 3 Student Research Committee, Afzalipour School of Medicine, Kerman University of Medical Sciences, Kerman, Iran; 4 State Key Laboratory for Zoonotic Diseases, Key Laboratory of Zoonosis Research, Ministry of Education, Institute of Zoonosis, College of Veterinary Medicine, Jilin University, Jilin, China; 5 Laboratorio Biología Molecular de Hidatidosis, Facultad de Medicina, Instituto de Microbiología Y Parasitología Médica (IMPaM), Consejo Nacional de Investigaciones Científicas Y Tecnológicas (CONICET), Universidad de Buenos Aires (UBA), Buenos Aires, Argentina

**Keywords:** *Echinococcus granulosus sensu stricto*, Canine echinococcosis, Dog intestine, Gene regulation, Transcriptome, Pepsin

## Abstract

**Background:**

*Echinococcus granulosus* sensu lato has a complex developmental biology with a variety of factors relating to both intermediate and final hosts. To achieve maximum parasite adaptability, the development of the cestode is dependent on essential changes in transcript regulation. Transcription factors (TFs) and miRNAs are known as master regulators that affect the expression of downstream genes through a wide range of metabolic and signaling pathways. In this study, we aimed to develop a regulatory miRNA-Transcription factor (miRNA-TF) network across early developmental stages of *E. granulosus* protoscoleces by performing *in silico* analysis, and to experimentally validate TFs expression in protoscoleces obtained from in vitro culture, and from in vivo experiments.

**Results:**

We obtained list of 394 unique *E. granulosus* TFs and matched them with 818 differentially expressed genes which identified 41 predicted TFs with differential expression. These TFs were used to predict the potential targets of 31 differentially expressed miRNAs. As a result, eight miRNAs and eight TFs were found, and the predicted network was constructed using Cytoscape. At least four miRNAs (egr-miR-124a, egr-miR-124b-3p, egr-miR-745-3p, and egr-miR-87-3p) and their corresponding differentially expressed TFs (Zinc finger protein 45, Early growth response protein 3, Ecdysone induced protein 78c and ETS transcription factor elf 2) were highlighted in this investigation. The expression of predicted differentially expressed TFs obtained from in vitro and in vivo experiments, were experimentally validated by quantitative polymerase chain reaction. This confirmed findings of RNA-seq data.

**Conclusion:**

miRNA-TF networks presented in this study control some of the most important metabolic and signaling pathways in the development and life cycle of *E. granulosus*, providing a potential approach for disrupting the early hours of dog infection and preventing the development of the helminth in the final host.

**Supplementary Information:**

The online version contains supplementary material available at 10.1186/s12864-023-09199-3.

## Background

One of the world’s most common zoonotic parasitic diseases is Cystic Echinococcosis or hydatid disease, causing remarkable health and financial burden in endemic countries [1]. The disease is caused by the dog tapeworm *Echinococcus granulosus* sensu lato, and is considered by the World Health Organization a neglected tropical disease (NTD). The life cycle of the parasite is going through the canid definitive host into the ruminant intermediate host in which the metacestode stage (hydatid cyst) is produced. Although humans are known to be accidental hosts, close human relationship with dogs and livestock, especially in endemic areas, is a challenge for disease control and prevention [2].

One of the critical stages in the life cycle of *E. granulosus* is the ability of the parasite to develop from the larval stage (protoscolex) to the adult stage (strobilated worm with mature proglottids) in the small intestine of the dog. However, due to the nature of *E. granulosus* life cycle, studies on the growth and development of the tapeworm in the final host are limited [3]. Therefore, exploring the cellular and molecular mechanisms involved in the strobilation process is still challenging. In recent years, a limited number of studies have been performed to characterize protoscoleces transcriptome and proteome in the presence of stimuli in the canine gastrointestinal environment [4–6]. The results of these studies highlight differentially expressed genes/miRNAs and newly synthesized proteins in the early stages of the strobilar development. These studies show the effects of environmental changes and the gastrointestinal stimuli on the evagination and strobilation of the parasite, however the underlying molecular/biological mechanisms have not yet been fully elucidated.

Transcription factors (TFs) and miRNAs are considered as the important master regulators, altering the expression of downstream genes in multiple pathways [7]. MiRNAs are small non-coding RNAs that regulate post-transcriptional gene expression. Normally, in animals a mature miRNA binds to the 3’ untranslated regions (3’UTRs) of the target mRNAs, thereby leading to mRNA degradation or protein synthesis suppression. A number of miRNAs has been identified in various *Echinococcus* species, related to the tissue and developmental stage specificity [8–11]. Even though, some common miRNAs could be detected in all *Echinococcus* species and their intermediate hosts [12].

Transcription factors control the rate of transcription by binding to the transcription factor binding sites (TFBS) in the enhancer or promoter regions of the target genes. However, depending on the type of transcription factor, the expression of the target genes can be increased or decreased [13]. Recent studies regarding the process of growth and development of *E. granulosus* have shown that changes in environmental factors can lead to extensive changes in transcriptome profiles in less than 24 h, including changes in the expression of TFs such as Forkhead box protein, Zinc finger protein 45, zinc-finger transcription factor Gli2 and Krueppel factor 10 [6, 14]. In another study on parasitic platyhelminths, some important TFs are predicted in the process of strobilation that co-expressed with other genes in Wnt, TGF-β/BMP, or G-protein coupled receptor signaling pathways [15].

Since miRNAs affect transcript stability, and TFs regulate mRNA production, a curious possibility of co-regulating shared target genes between miRNAs and TFs could be expected. In the last decade an increasing number of studies have been focused on the association of miRNAs and TFs in the pathogenesis of human diseases including leukemia, cleft lip, hepatic cancer stem cells and testicular germ cell tumors [16–20]. In infectious diseases, such as Covid-19 and TB, regulatory linkages between TFs and miRNAs have recently been discovered [21, 22]. Therefore, it would be promising that understanding miRNA-TF network could lead to identifying key regulatory motifs and cellular regulatory mechanisms in echinococcosis. As there are limited studies in the miRNA-TF network analysis in parasitic helminths, particularly cestode parasites, the identification and characterization of these networks can improve our knowledge on the factors affecting parasite development and pathogenic mechanisms.

Comparative analyses of *E. granulosus* transcriptome and miRNAome profiles in different developmental stages are scarce. Also, the value of in vitro development of the strobilated worms as an alternative to in vivo experiments in dogs is not fully evaluated and studies on the phenomenal behavior of *E. granulosus* development in in vitro culture compared to early development of the parasite in the natural definitive host is still challenging. In this study, we attempted to construct a regulatory miRNA-TF network across in vivo and in vitro early developmental stages of *E. granulosus* protoscoleces.

## Results

**Fig. 1:**
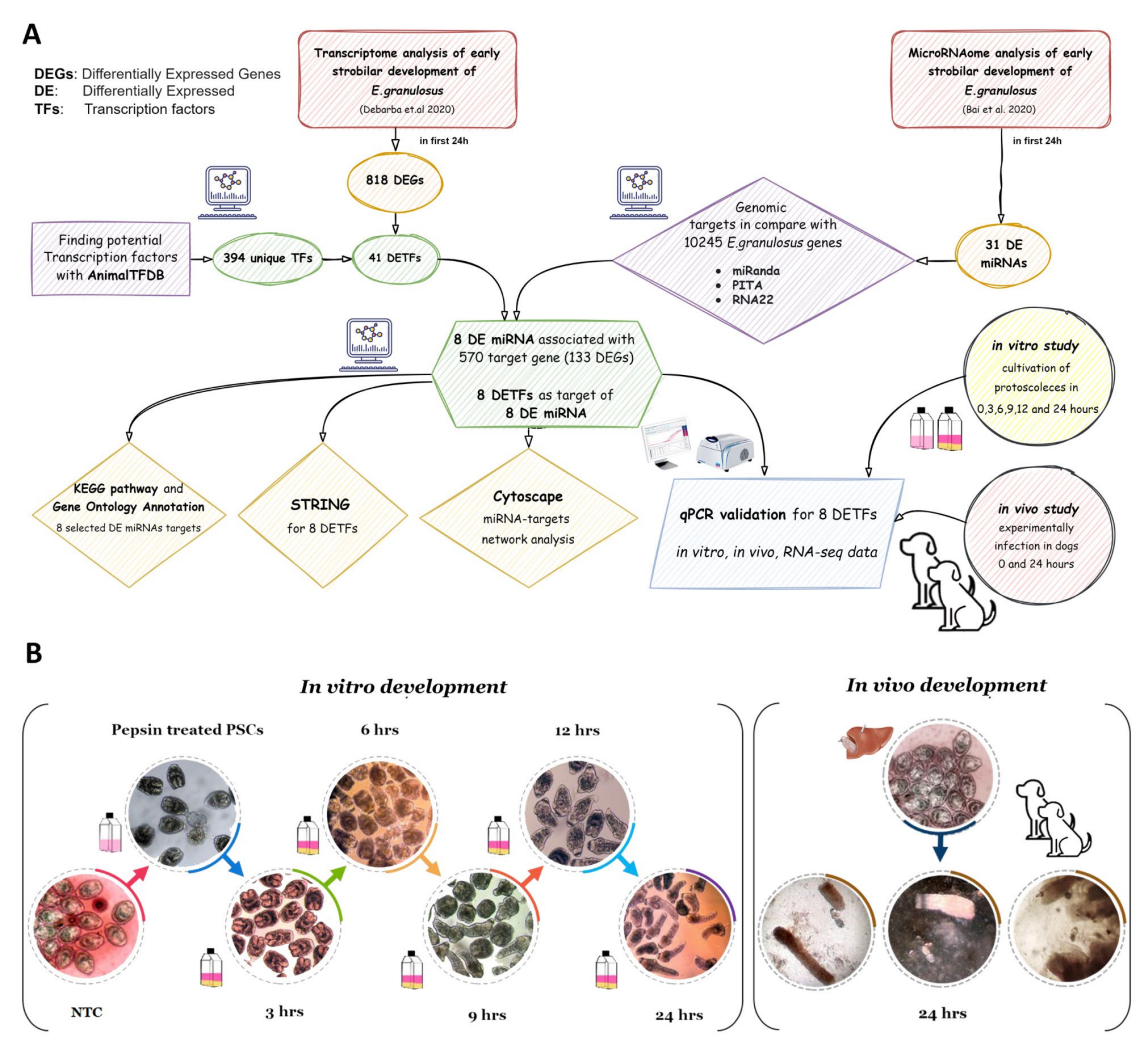
** A.** The workflow and the summery of findings of miRNA–TF network analysis in the early strobilar development of *E. granulosus* protoscoleces. **B.** An overview of the comparative experimental study on the early strobilar development of *E. granulosus* protoscoleces; In vivo and *in vitro settings*

Figure 1 A explains the workflow of this study to construct comprehensive miRNA–TF networks in the early strobilar stage of *E. granulosus* by using *in silico*, in vitro and in vivo analyses. In vivo and in vitro development experiences was successfully performed for all time dependent treatments. In vitro pepsin treated *E. granulosus* protoscoleces were cultivated for 0, 3, 6, 9, 12 and 24 h and collected along with non-treated controls (Fig. 1B). For in vivo experiments, early strobilar stages harvested from dogs’ intestine were compared with the protoscoleces samples from the same cyst initially fed to the dogs. In vitro culture showed a gradual development during the first 24 h after pepsin treatment. In our in vivo development experiments, similar to in vitro conditions, 24 h after dog infection, evaginated protoscoleces developing towards strobilar worms were visible. Figure 1. **B** shows the worms in the very beginning of the parasite settlement in the proximal sections of the dog intestine.

### Identification of differentially expressed transcription factors

**Fig. 2:**
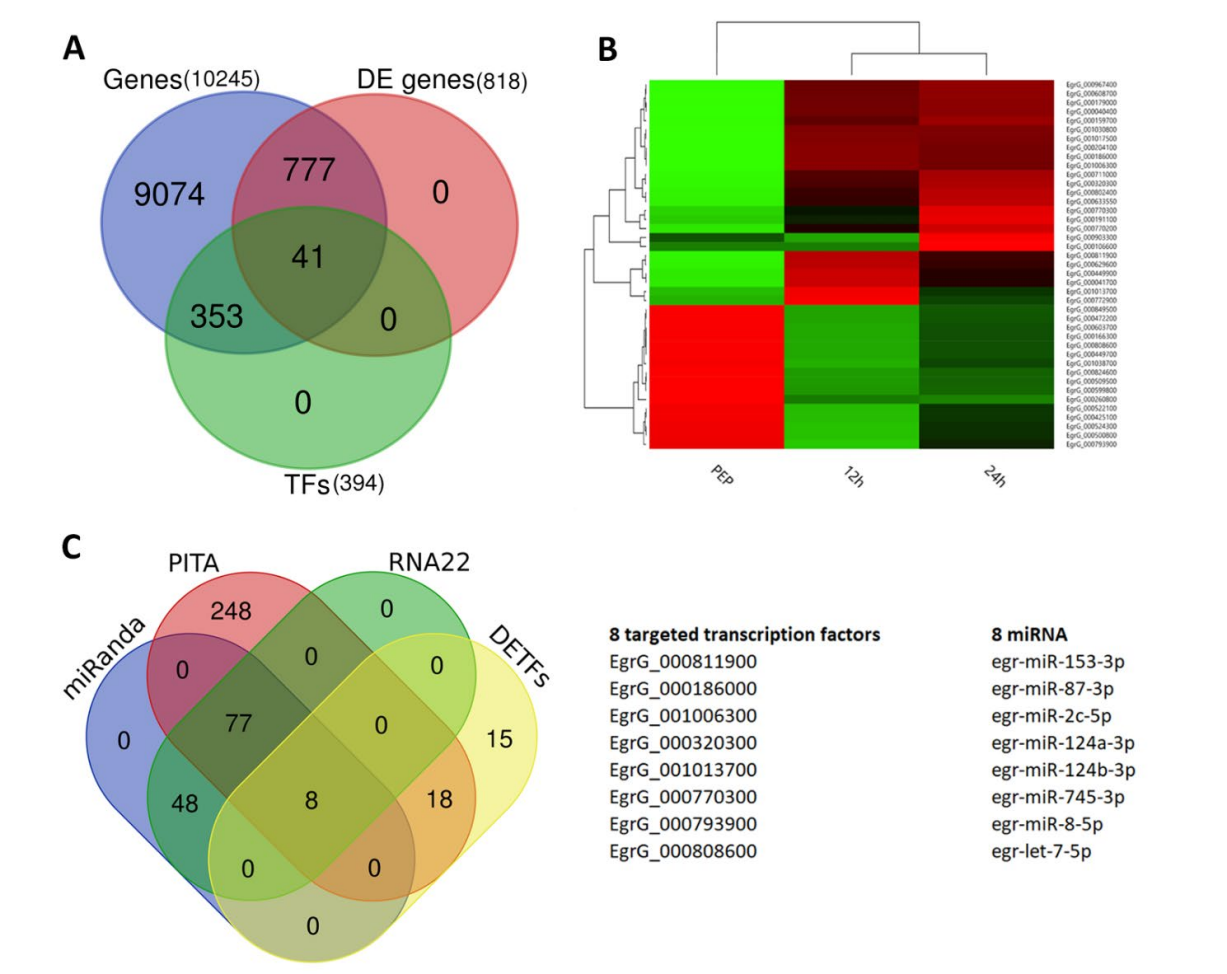
** A.** The identification of 41 differentially expressed transcription factors (DETFs) based on comparison of 10,245 *E. granulosus* genes, along with 394 of *E. granulosus* transcription factors (TFs) and 818 differentially expressed genes in the early strobilar development of *E. granulosus* protoscoleces. **B.** Heatmap clustering of 41 differentially expressed TFs of *E. granulosus* according to RNA-seq data (Debarba et al., 2020). Pepsin-treated protoscoleces (*PEP, 0 h)*, Pepsin-treated protoscoleces after 12 *(12 h)* and 24 h *(24 h)*. The Z-score calculation on fold changes was utilized. **C.** Identification of eight differentially expressed TFs and corresponding miRNA targets in comparison to a list of 41 DETFs with predicted targets from miRanda, PITA, and RNA22.

Among 10,245 protein-coding genes of *E. granulosus* (PRJEB121), 394 potential TFs were identified and classified into 58 families by using AnimalTFDB database. The highest number of TFs belonged to ZF-C2H2 (82), Homeobox (58), bHLH (31), ZF-C2H2_2 (26) and HMG (22) families, respectively. The comparison of these potential TFs with 818 differentially expressed genes (DEGs) in the early strobilar development of *E. granulosus* protoscoleces showed 41 TFs with differential expression (DETFs) (Fig. 2A, Additional file 1: Table S1). The expression level of co-clustered TFs in 12- and 24-hour treatments showed higher correlation coefficients compared to the pepsin treated samples (Fig. 2B).

### Target gene prediction and network analysis of miRNA-target genes

**Fig. 3:**
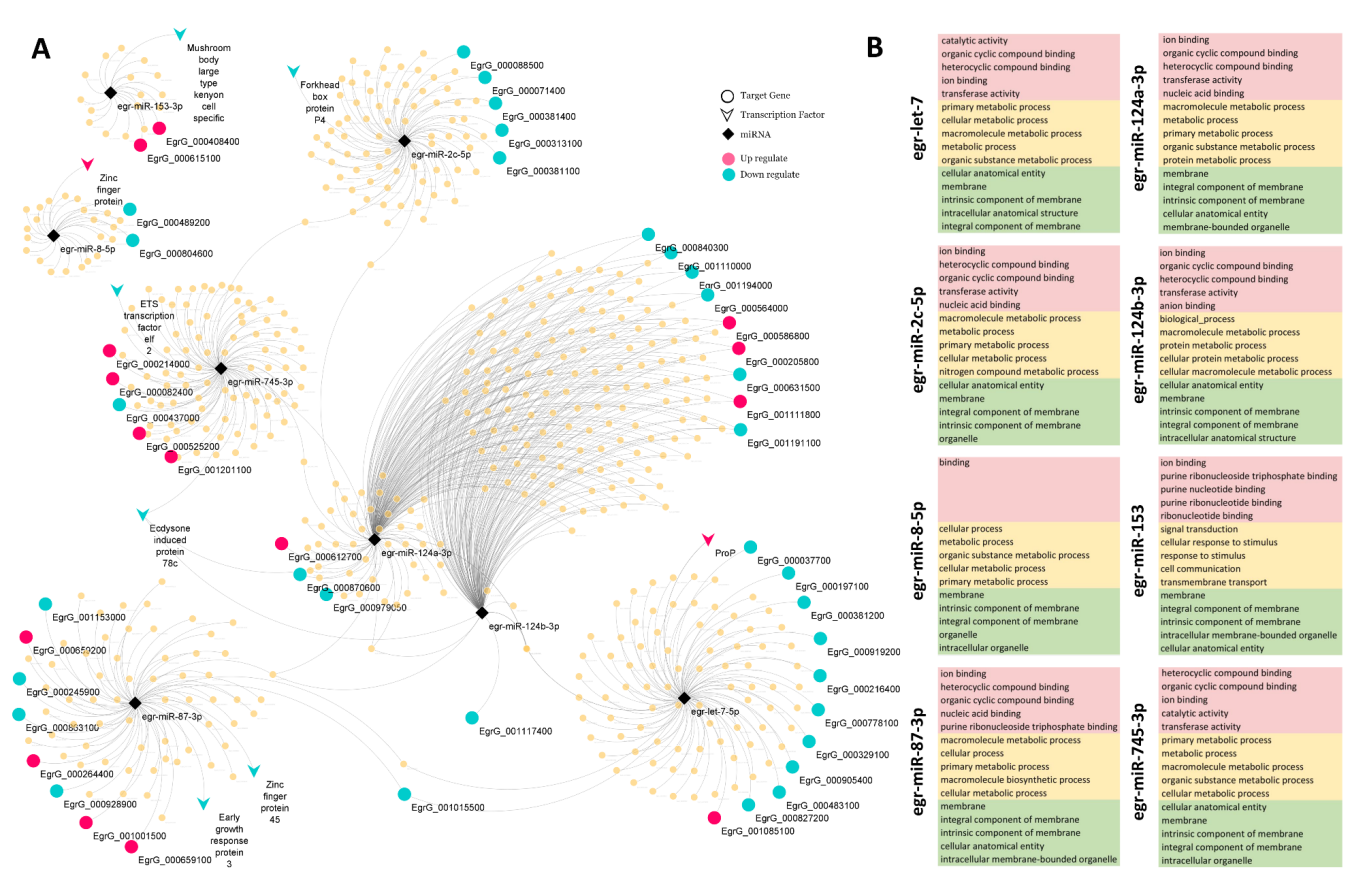
** A.** miRNA–target interaction networks of Echinococcus granulosus genes with differential expression in early strobilar development of protoscoleces. **B.** Gene Ontology enrichment of differentially expressed genes related to selected miRNAs. The enrichment analysis of the cluster of related genes to each miRNA separated into three categories: Molecular Function (red), Biological Process (yellow) and Cellular Component (green)

We focused on master regulatory elements, miRNA-TF network in the early 24 h of developmental stages of *E. granulosus.* Eight miRNA-target genes were selected by comparing the outcome of three popular miRNA interaction predictor algorithms. The results of miRanda, PITA and RNA22 were summarized in Fig. 2. **C** and Additional file 1: Table S2. To make the miRNA-target genes interactions more specific for miRNA-TF relation, we limited the predicted targets to the list of 41 TFs with differential expression in the early strobilar development. Consequently, eight miRNAs and eight TFs were identified and indicated in Fig. 2.C and Fig. 3. **A**. In this regard, each TF was related to one, two or three miRNAs. The constructed network with 578 nodes and 722 edges were analyzed by Cytohubba and the egr-miR-124a-3p was indicated as top ranked in Degree/Closeness/and Betweenness centrality. Accordingly, the target genes of eight miRNAs were clustered in three subnetworks (Fig. 3. **A)**.

### GO function and KEGG pathway enrichment

Functional enrichment analysis of 570 putative targeted genes of eight selected DEmiRNAs identified several enriched GO terms in cellular component, biological process and molecular function. The cluster of target genes to each miRNA were analyzed separately and the top ranked GO terms in each cluster were summarized (Fig. 3. **B)**. The ion binding (GO:0043167) and heterocyclic compound binding (GO:1,901,363) are the most significant GO terms observed in each cluster genes in Molecular Function category. The membrane (GO:0016020), integral/intrinsic component of membrane (GO:0031224, GO:0016021) and cellular anatomical membrane (GO:0110165) are the most frequent and significant GO terms observed across all clusters in cellular components. The biological process is the most diverse category, with all targeted genes. However, the GO terms associated with metabolic processes are more prominent in each target gene cluster. The findings of the gene cluster analysis for each miRNA are provided in the **Additional file 2.**

According to the annotation level of the *E. granulosus* genome, the possibility of identifying KEGG Orthology (KO) for all target genes was limited. Nonetheless KEGG pathway analysis indicates that crosstalk between each miRNA and the related targeted gene were much multifaceted (**Additional file 1. Table S3**). The Metabolic pathways and Biosynthesis of secondary metabolites are the most Frequent pathways through all miRNA’s targets. In this regard, Glucagon signaling pathway, Starch and Sucrose metabolism and Insulin signaling pathway were the highlighted Enriched pathways in targeted genes of egr-miR-2c-5p. Interestingly, egr-miR-124a-3p, egr-miR-124b-3p, egr-miR-745-3p and egr-miR-87-3p showed a significant relationship with the splicing pathway and specifically to spliceosome complex (**Additional file 1. Table S3**).

The Longevity regulating pathway was another common shared pathway among egr-let-7-5p, egr-miR-745-3p, egr-miR-8-5p and egr-miR-124a-3p. Also, A number of vital pathways in cell regeneration and development such as Axon regeneration, Apelin signaling pathway, signaling pathways regulating pluripotency of stem cells were found in related to each cluster of targeted genes. Hippo signaling pathway, an evolutionarily conserved signaling pathway controlling organ size in animals, was the common pathway among egr-miR-124a-3p, egr-miR-124b-3p, egr-miR-87-3p and egr-miR-745-3p (**Additional file 1. Table S3**).

### Functional enrichment analysis of transcription factors interaction networks

Searching the recurring instances of neighboring genes with STRING provides a predicted protein − protein interaction/association network for six TFs as hub genes. The transcription factor, early growth response protein 3 (EgrG_001006300) as hub gene was related to some of the well-known genes regulating DNA expression such as histone binding protein and histone deacetylase. This TF along with Zinc finger protein (EgrG_000793900) and Zinc finger protein 45 (EgrG_000186000) have a role as gene hub with Mind bomb and some polymerases that catalyze the transcription of DNA into RNA. Interestingly, the Ecdysone induced protein 78c (EgrG_001013700) as gene hub show a relation with Forkhead box protein P4 (EgrG_000320300), another differentially expressed TF in this study. The results of the predicted networks for each TF are listed in **Additional file 1. Table S4**.

### Validation of transcription factor expression in the protoscoleces from in vitro and in vivo settings

**Fig. 4:**
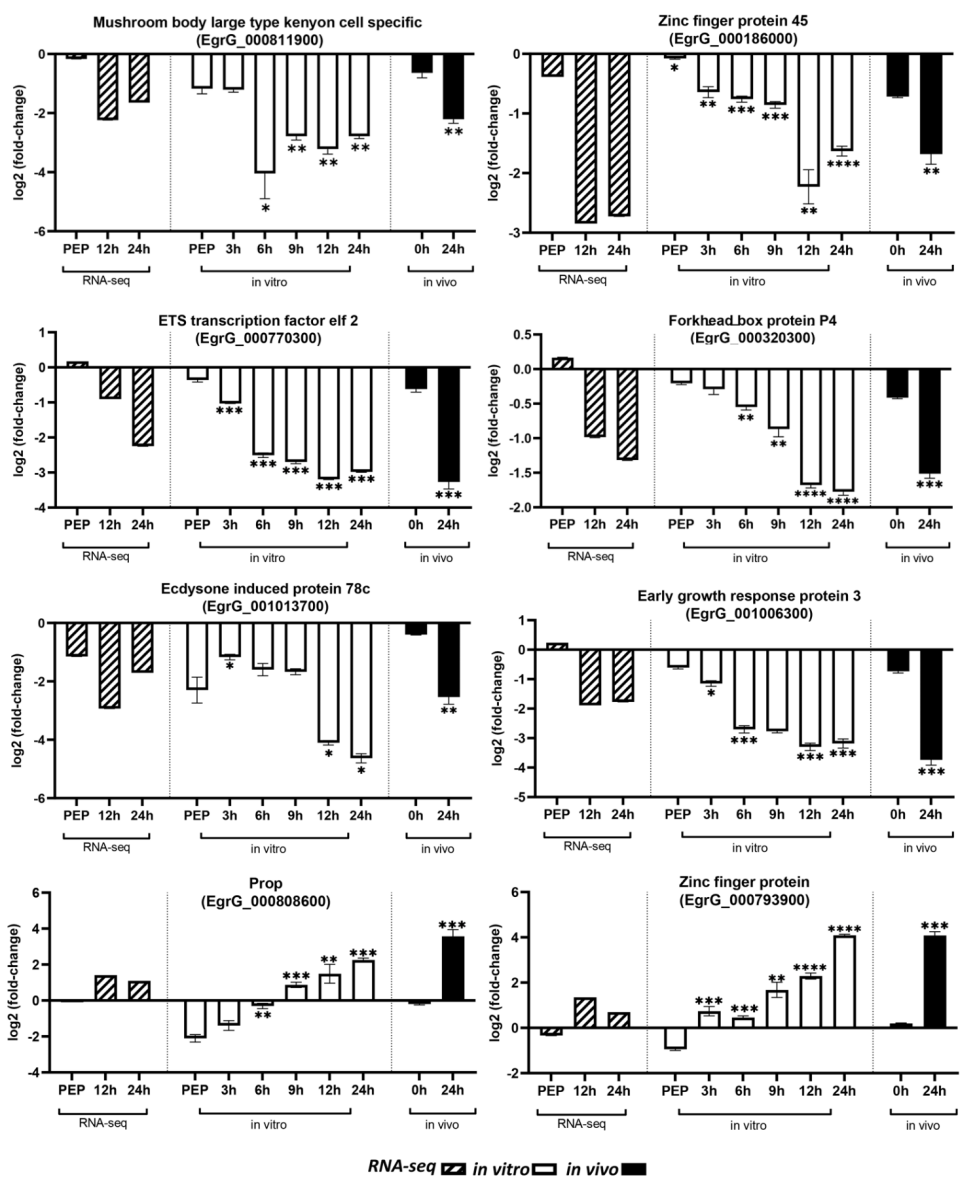
Expression of eight predicted transcription factors differentially expressed in early strobilar development of *Echinococcus granulosus* in in vitro and in vivo experiments compared to RNA-seq data. The in vitro test groups were analyzed in comparison with PEP group. * Statistically significant, P < 0.05

We validated the expression profiles of eight differentially expressed TFs at different time points of early strobilar development of *E. granulosus* protoscoleces from in vitro and in vivo samples (Fig. 4). In the in vitro experiment, the expression of six TFs was gradually reduced over the first 24 h of early strobilar development. These results were consistent with the expression data from the collected early developed protoscoleces in our in vivo experiment and previously published RNA-seq data.

Over the same time period, the expression of the other two TFs (EgrG 000793900, EgrG 000808600) steadily increased in both in vivo and in vitro study groups. These findings followed a similar pattern to previously published RNA-seq data. (Fig. 4).

## Discussion

In the present study we tried to improve the understanding of miRNA and TF regulatory networks in the early developmental stages of *E. granulosus* protoscoleces which are essential in parasite reproduction and development in the final host as well as in the in vitro conditions. The process of evagination of protoscoleces in the dog intestine is a critical stage of *E. granulosus* development toward strobilation, but the logistical, ethical, and safety issues associated with conducting in vivo experiments on *Echinococcus* definitive hosts are major limiting factors in research on the adult worm development [3]. After ingesting hydatid cysts, the protoscoleces are released from brood capsules by pepsin action, and the adult worm development processes start in dog gastrointestinal tract [23, 24]. Although it was possible to feed collected protoscoleces to dogs, in this study the intact cysts were fed to the dogs in order to mimic the natural cycle of infection [25]. Considering that the effect of combined enzymatic digestion and bile salts in stimulation of strobilation has been shown before, the same experimental settings as performed in the previous RNA-seq studies, were applied in the culture of protoscoleces [4, 5]. Protoscoleces are particularly active during and after evagination because they need to quickly settle in the GI tract of the definitive host in order to prevent being washed out of the small intestine. Protoscoleces motility is facilitated by a well-developed neural system and glycogen energy reserves that are rapidly used in the early developmental stages. Although this process might take up to 72 h, most protoscoleces evaginate and establish within six hours after ingestion [3, 14, 26].

It is critical for the protoscoleces to use advantageous strategies in the early hours of evagination in order to adapt quickly and flexibly to changes in definitive host intestinal environment. [23, 24]. TFs are well-known for their roles in response to environmental stimuli and helminth development, therefore we put together a list of 394 *E.* granulosus TFs and compared them with 818 DEGs. As a result, the analysis discovered 41 TFs with differential expression.

A recent study indicates that *E. granulosus* has more alternatively spliced genes than other platyhelminths, suggesting the parasite’s need to adapt to various settings within the bodies of intermediate and definitive hosts and highlighting the function of regulatory mechanisms of gene expression [14]. The regulatory network of miRNAs and TFs, as two key master regulatory elements, might have an impact on the survival of *E. granulosus* during the first 24 h of development. Therefore, we compared 818 differentially expressed genes of *E. granulosus* previously identified by Debarba 2020 with 31 differentially expressed miRNAs in the early stages of protoscoleces previously identified by Bai et al. [5, 6].

In the current study, we discovered a significant shift in the regulatory networks of multiple differentially expressed miRNAs and TFs, regulating key pathways in parasite survival in the first 24 h after ingesting hydatid cysts by the definitive host. We applied three well-known miRNA interaction prediction algorithms with different approaches for target identification; i.e., RNA22, PITA, and miRanda. Finally, the predicted targets in each software were compared to the DETFs list, and eight DEmiRNAs with putative effects on eight DETFs in the early stages of strobilar development were selected. These findings well matched with previously published miRNA expression data from the same period [5]. As a result, the transcription factor targets of 6 out of the 8 upregulated miRNAs were downregulated in the in vivo and in vitro experiments **(Additional file 1. Table S5)**. This miRNA expression pattern is consistent with the basic concept of gene expression regulation by miRNAs: as miRNA expression increases, the expression of its target genes decreases. However, this trend is different for Zinc finger protein (EgrG_000793900) and ProP (EgrG_000808600). Moreover according to Bai et al. the amount of fold change in egr-miR-8-5p during the first 24 h of the strobilation stage in the original research was not remarkable [5]. It is probable that the relationships between the target gene and the miRNA in these two cases have not been accurately predicted, even if there may be other regulatory variables e.g., lncRNAs, acting on these TFs and/or miRNAs. However, in other conditions or life cycle stages, these genes might nevertheless be targeted by miRNAs.

In the proposed miRNA-TF network, it is interesting to see that certain TFs are affected by multiple miRNAs and a single miRNA controls a number of TFs. The ecdysone induced protein 78c (EgrG_001013700) is related to three miRNAs, egr-miR-124a-3p, egr-miR-124b-3p and egr-miR-745-3p. The *C. elegans* ortholog of this gene (Signal Element on X, sex1) is involved in primary sex determination and the regulation of gene expression [27]. Also, The Ecdysone-Induced protein 78c is one of the characterized nuclear receptors in the parasitic platyhelminth, *S. mansoni* [28]. Among the three miRNAs listed, egr-miR-745-3p also regulates the expression of the ETS transcription factor elf2 (EgrG 000770300). The orthologous *C. elegans* elf transcription factor is predicted to mediate DNA-binding transcription factor activity, specific RNA polymerase II, regulate cell division and be involved in gonad development [29]. ETS like transcription factor as calcium sensitive TFs has been previously identified in larval stage of *E. granulosus* [30]. As a result, it appears that changes in these regulatory factors can have a significant impact on the growth of new proglottids and the formation of reproductive organs.

On the other hand, while the egr-miR-745-3p regulates both ecdysone-induced protein 78c and ETS transcription factor elf2, the egr-miR-87-3p regulates Zinc finger protein 45 (EgrG_000186000) and Early growth response protein 3 (EgrG_001006300). The Early growth response protein 3 has protein-protein association networks to some important protein in development and cellular proliferation such as mind bomb and histone deacetylase. The *C. elegans* mind bomb ortholog (mib-1) is predicted to have ubiquitin-protein transferase activity and predicted to participate in the Notch signaling pathway, which is important for normal embryonic development and tissue homeostasis through regulating a number of cell fate decisions and cellular processes [31]. Also, The Notch signaling system is important in nephron segmentation and formation and function of the nervous system [32, 33]. Notably, the elevated expression of Notch pathway-related genes in the peripheral blood of persons with *S. haematobium* was reported in another investigation as a possible indication of the systemic inflammation associated with chronic schistosomiasis [34]. As a result, it appears that the sequential regulatory process of miRNAs and TFs plays a key role in cellular differentiation and the formation of body organs in the parasite life cycle, ultimately influencing the process of protoscolecses development to adult worms.

As it mentioned earlier, Protoscoleces appears to be responding to environmental changes in the host’s intestine by significantly changing its transcriptome and proteome levels which is consistent with the worm’s preparation for the strobilation as well as the process of providing the required energy. According to the recent investigations including the present study, this response is identified to occur efficiently as a swift and quick process [4, 6, 14]. The KEGG pathway enrichment of each clustered DE gene in the constructed network shows that egr-let-7-5p, egr-miR-2c-5p, egr-miR-124a-3p, egr-miR-124b-3p, egr-miR-745-3p, and egr-miR-87-3p regulate several genes involved in the metabolic and biosynthesis of secondary metabolites pathways. In particular, we should highlight the function of egr-miR-2c-5p in regulating the metabolic pathways involved in energy intake, such as starch and Sucrose metabolism (ko00500), insulin signaling (ko04910), and glucagon signaling pathway (ko04922) by regulating number of genes such as EgrG_000491400, EgrG_000501500, and EgrG_001087500. It has already shown that the evaginated protoscoleces hold some glycogen resources, which serve as an energy reserve, but it is quickly used within a few hours, and protoscoleces need to look for other sources of energy [35]. Interestingly, EgrG 000491400 (Calmodulin - K02183), one of the best-known genes for ion binding and calcium-mediated signaling throughout the life cycle of cestodes, is targeted by egr-miR-2c-5p. Our findings on the GO enrichments of each cluster of miRNA-targeted genes indicate that ion binding and membrane roles are more prominent, which can be due to the organism’s requirement to react properly to environmental changes. In accordance to our findings in a recent study on *Fasciola hepatica*, the expression of integral component genes of the membrane was found as the main biological function in the newly excysted juvenile [36].

Considering the limited number of genes in *E. granulosus*, it seems that the presence of an active splicing mechanism plays an essential role in cestode adaptability [14]. Interestingly, at least four selected miRNAs (egr-miR-124a-3p, egr-miR-124b-3p, egr-miR-745-3p and egr-miR-87-3p) control the spliceosome complex genes including heat shock 70 kDa protein (*HSPA1s*), U4/U6 small nuclear ribonucleoprotein (*PRP31*), U4/U6.U5 tri-snRNP-associated protein 2 (*USP39*), pre-mRNA-splicing factor (*SYF2*), U6 snRNA-associated Sm-like protein (*LSM3*), FUS-interacting serine-arginine-rich protein(*FUSIP1*), ATP-dependent RNA helicase (*DDX23*) and splicing factor, arginine/serine-rich 7 (*SFRS7*). These findings are in line with the results of a previous study showing that alternative splicing is frequently occurring in spliceosomes complex during the early stages of the *E. granulosus* strobilation and emphasize the significance of the splicing machinery in the cestode development [14].

It is crucial to note that egr-miR-124a-3p has the highest rank of communication degree when compared to other miRNA nodes in the present miRNA-targeted genes network, including selected DETFs (**Additional file 1. Table S6**). This miRNA targets a number of genes involving in important energy intake related pathways such as metabolic pathways, biosynthesis of secondary metabolites, galactose metabolism, insulin signaling pathway, glycolysis/gluconeogenesis and some other critical pathways in the life cycle of *E. granulosus* such as Spliceosome, MAPK signaling, focal adhesion, Hippo signaling, and longevity regulating pathway. The Hippo signaling pathway, which regulates organ size by controlling cell proliferation, apoptosis, and stem cell self-renewal, is likewise targeted by egr-miR-124b-3p, egr-miR-745-3p, and egr-miR-87-3p. This is confirmed by a previous investigation on the miRNA profile of *E. granulosus* strobilated worms generated from in vivo and in vitro systems, which revealed that differentially expressed miRNAs were implicated in Hippo signaling and MAPK pathways [37]. It is remarkable that egr-miR-124a-3p also targets some genes involving in the longevity regulating pathway along with egr-miR-745-3p egr-miR-8-5p and especially egr-let-7-5p. This would lead to the conclusion that the selected miRNAs network in particular egr-miR-124a-3p, plays a critical role in the protoscoleces ability to adapt to environmental changes and to prepare for the next stage of the tapeworm life cycle.

Findings of the present study have potential implications for CE prevention and control. Due to the nature of *E. granulosus* biology in the final host, there is currently no reliable prevention method for echinococcosis in dogs. Our results suggested that the proposed miRNAs of the miRNA-TF network might be potential targets for canine vaccinations. Given the recent success of RNA-based immunizations in the fight against COVID-19, this technology could be used to specifically target miRNAs [38–40]. It is also assumed that disrupting the predicted miRNA-TF network can be achieved by modifying the intestinal environment. Several studies successfully used host microbiome manipulation, as a non-invasive approach, to disrupt miRNA regulatory function [41, 42]. This provides a potential for using this approach to disrupt miRNA-TF network particularly in the early hours of infection before the protoscoleces settled in dog intestine, preventing development of the helminth in the final host.

Despite many efforts made in the last decade to enrich our knowledge on *E. granulosus* genomics, transcriptomics and proteomics, the databases on the “neglected neglected” cystic echinococcosis still have very limited annotations. In the present study, like many other studies in this field, the data analysis, and the results were highly dependent on the information obtained from other platyhelminth species such as *Schistosoma spp*. or nematodes such as *C. elegans*.

## Conclusions and outlook

**Fig. 5:**
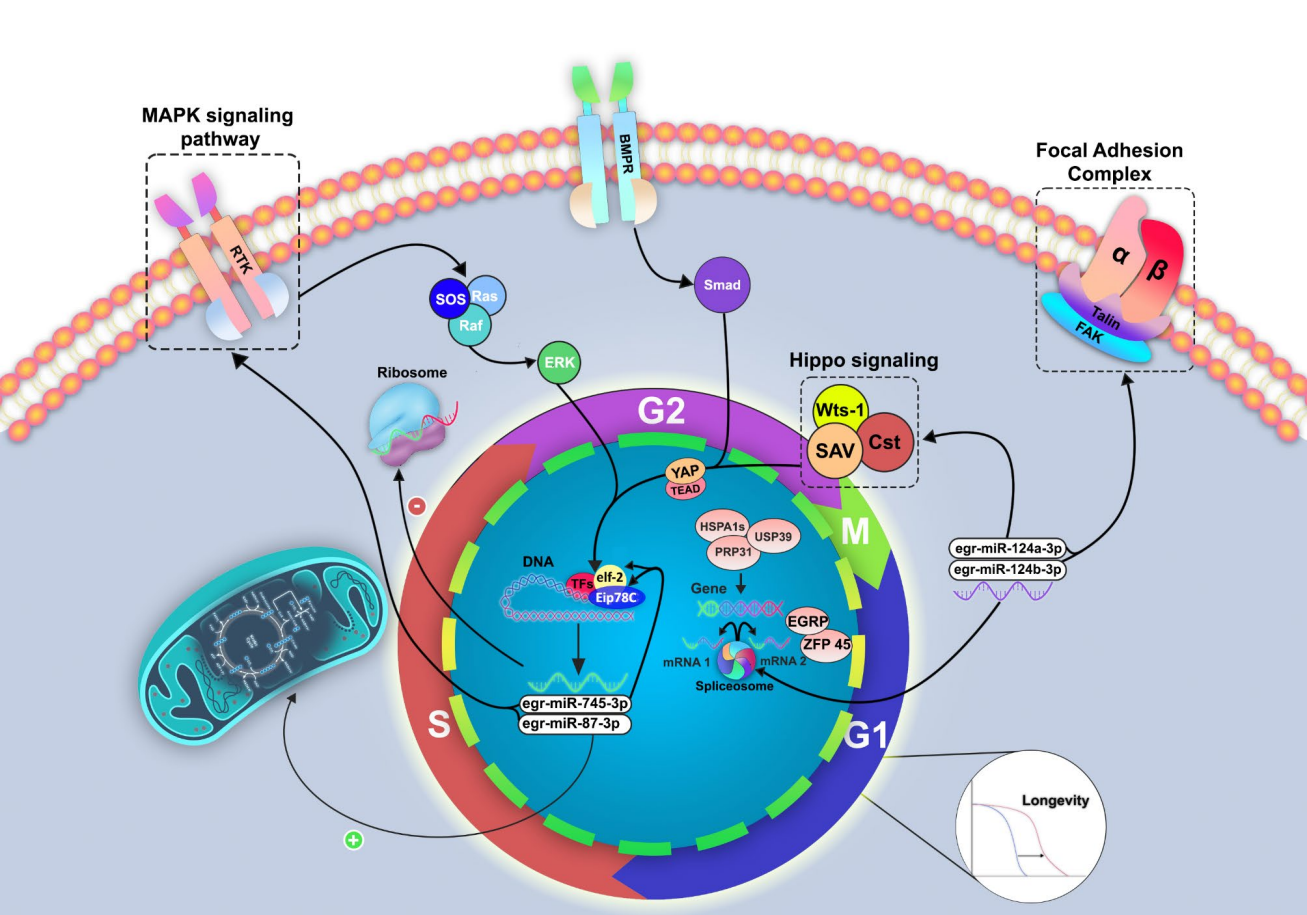
Schematic diagram of the hypothetical role of transcription factors, miRNAs and their related cell pathways/functions to the survival and development of *Echinococcus granulosus* in early hours of dog infection. The set of four differentially expressed miRNAs (egr-miR-124a-3p, egr-miR-124b-3p, egr-miR-745-3p and egr-miR-87-3p), were mapped into different cell signaling pathways and assigned to different functional processes in early cestode housing and strobilation. These miRNAs target some transcription factors that plays a key role in cellular differentiation and the formation of body organs in the parasite life cycle

The present study demonstrates the nature of miRNA-TF regulatory networks in the early developmental stages of *E. granulosus* protoscoleces in the definitive host as well as in the in vitro conditions. In the absence of information on the tapeworms, we believe that the data sets can be used as an initial assessment of the relationship between transcription factors and miRNAs. However, further studies are required to investigate regulatory networks in more comprehensive investigations in a bi-directional setting. We highlighted at least four miRNAs and their targeted TFs differentially expressed in the first 24 h of dog infection. These master regulatory elements control some of the most important metabolic and signaling pathways in the development and life cycle of the parasite (Fig. 5). Based on the current findings, future efforts toward designing targeted drugs and vaccines against canine echinococcosis specifically in the early hours of infection and disrupting the parasite life cycle. Also, it is essential to proceed with further studies to enrich our knowledge of the current *E. granulosus* omics databases to provide more precise predictions.

## Methods

### Parasite material

Hepatic hydatid cysts from naturally infected sheep were collected from local municipal abattoir. All specimens were transferred to the research center for hydatid disease in Iran and the viability and motility of protoscoleces in each cyst was determined by eosin exclusion test and light microscopy. Genotyping of the isolates were performed using PCR-sequencing based on J. Bowles cytochrome c oxidase primers [43] and *E. granulosus* sensu stricto G1 genotype was used in the study. Protoscoleces with more that 90% viability were used for further in vivo and in vitro experiments.

### In vivo development

Two mix-breed, 3 to 6 months old, female dogs were provided by a local vendor. After adapting to the new environment, the animals were treated with 10 mg/kg GUADREKS, Vilsan Veterinary Pharmaceuticals (Each tablet contains 250 mg Praziquantel). All the fecal materials were collected and disposed according to safe disposal instructions. To create the least difference compared to the natural cycle of infection in the final host, both dogs were experimentally infected by feeding cysts in one piece. A portion of the protoscoleces was kept as non-treated control for gene expression comparisons. Twenty-four hours after infection, the animals were euthanized according to the reference manual guideline [44]. After dissection, the small intestine was divided into 20-cm long segments and each segment was scraped and examined under a stereomicroscope. Each segment was explored in the individual 30-cm transparent dishes and four researches work individually on each segment. The surface of the intestine was first scraped and its content was precipitated in less than 10 min. Then, the sediment was transferred to a 10-cm Petri dish by using a pipette, and the evaginated protoscoleces were isolated with a micropipette tip. It is realized from the findings of our previous study that a higher density of the worms can be expected in the proximal segments of the jejunum [45]. Therefore, in this study, more focus was made to find protoscoleces faster in these areas. In less than an hour, evaginated protoscoleces moving in intestinal microvilli, were collected using a micropipette and washed with normal saline. The protoscoleces were preserved in liquid nitrogen until use.

### In vitro development

In vitro culture of protoscoleces was carried out as previously described for investigating early strobilar development of *E. granulosus* in triplicates with minor modifications [4, 5, 14]. Briefly protoscoleces from sheep liver hydatid cyst were washed three times in phosphate-buffered saline (PBS), and incubated for 15 min with pepsin (2 mg/mL, pH 2.0) at 37 °C and subsequently the PSCs were cultivated in biphasic medium containing fetal bovine serum (FBS) and RPMI 1640. The solid base of the medium was made by heating newborn calf serum at 76◦C for 45 min and the liquid phase was RPMI 1640 medium with 20% (v/v) fetal calf serum, 0.45% (w/v) yeast extract and 0.4% (w/v) glucose. The protoscoleces were cultured at 37◦C and 5% CO_2_ in the above medium containing 0.02% (w/v) dog bile. At 0, 3, 6, 9, 12 and 24 h after incubation protoscoleces were harvested. Pepsin-free treated protoscoleces were used as controls. Cultured flasks were placed in an upright position in the incubator at 37 °C with 5% CO_2_.

### Data resources

Transcriptomic data were collected from two recently published studies on the early strobilar developmental stages of protoscoleces. All differentially expressed genes (DEGs) at the first 24 h were retrieved from Debarba et al. 2020 **(Additional file 1. Table S7)**[6]. Likewise, differentially expressed miRNA data at the first 24 h of protoscoleces development were collected from Bai et al. 2020 **(Additional file 1. Table S8)**[5].

### Exploring potential transcription factors

For identifying potential TFs we used AnimalTFDB [46], as a well-known comprehensive database for prediction and classification of genome-wide TFs. In this regard, the peptide sequences of *E. granulosus* of genome project PRJEB121 were collected from the WormBase ParaSite using the Biomart tool, and subsequently submitted to the online database AnimalTFDB 3.0 (http://bioinfo.life.hust.edu.cn) to identify all possible TFs in the genome. Transcription factor family classification was performed by the default settings in AnimalTFDB algorithm **(Additional file 1. Table S9)**. To identify TFs with differential expression, the predicted TFs of *Echinococcus* was compared to DEGs and the final list was used for future analyses (Additional file 1 Table S1).

### Exploring miRNA-mediated gene/ transcription factor regulation

MiRanda [47], RNA22 [48] and PITA [49], three popular miRNA interactions predictor algorithms, were used for exploring putative miRNA targets. The sequences of selected miRNAs of *E. granulosus* differentially expressed at the first 24 h of developmental stage **(Additional file 1. Table S8)** were collected from miRbase (http://www.mirbase.org/). Also, 300 nucleotides were extracted downstream from the stop codon of each *E. granulosus* coding gene (genome project PRJEB121) using BioMart tool. The miRanda parameters used were: (i) strict seed pairing; (ii) score threshold: 140; (iii) energy threshold: -20 kcal/mol, (iv) gap open penalty: -9; (v) gap extend penalty: -4; (vi) scaling parameter: 4 according to previous study [45]. The PITA settings were applied to the default computation workflow with few modifications and targets with ΔΔG less than or equal to -10 kcal/mol were selected. The preset parameters were utilized to apply RNA22. The consensus targeted genes predicted by the three algorithms were compared with lists of DEGs in early strobilar developmental stage and the related miRNA-target genes including TFs with differential expression were recognized.

### Network and subnetwork analyses and functional evaluation

The miRNA–mRNA network of targeted genes were visualized by using Cytoscape (version 3.8.2) [50] for selected miRNAs. We also visualized and analyzed the topological properties of the regulatory networks and identified hubs using the Cytoscape plugin, yFiles Layout. The subnetwork of miRNA-TFs was constructed by comparing target genes with lists of DEGs in early strobilar developmental stage. Based on Degree centrality, Closeness centrality and Betweenness centrality, the cytoHubba (version 0.1) [51] was used to find the key functional modules and the top ranked miRNAs/Genes in the network. The clusters of each group of miRNA target genes were compared individually for gene ontology and pathway enrichment analysis.

### GO function and KEGG pathway enrichment analysis

Functional annotations and gene ontology (GO) of each cluster of miRNA target genes were performed by using BLAST searches against the NCBI non-redundant protein and InterPro scan databases by using OmicsBox software [52]. The online analysis toolkit WEGO 2.0 [53] and gProfiler web server [54] were used to identify the overrepresented GO terms within each clustered miRNA target genes of *E. granulosus*. The Benjamini-Hochberg procedure was applied to adjust the false discovery rate (adjusted p-value threshold < 0.05). KEGG Automatic Annotation Server (KAAS) was used for comparing the transcripts of each clustered miRNA target genes for ortholog assignment and pathway mapping [55].

### Functional enrichment analysis of protein-protein interaction networks

Protein-protein interaction networks for each selected TF were visualized using the Search Tool for Recurring Instances of Neighboring Genes (STRING) [56]. The active interaction sources for search criteria was limited to Experiments, Databases, Co‑expression, Neighborhood, Gene Fusion and Co‑occurrence and minimum required interaction score was set on the medium confidence 0.400.

### Validation of TF expression in the protoscoleces from in vitro and in vivo settings

Total RNA was extracted from in vivo and each replicate of in vitro samples at different time points using YTzol Pure RNA (Yekta Tajhiz Azma, Iran), according to the manufacturer’s instructions. RNA concentration and purity were measured using 260/280 and 260/230 ratios with a spectrophotometer (NanoDrop ND-2000; Thermo Fisher Scientific, Inc., Wilmington, DE, USA).

An equal amount of all isolated RNAs were used to synthesize cDNA by using Transcriptor First Strand cDNA Synthesis Kit (Roche, Germany) with appropriate random hexamer primer in a final volume of 20 µL according to the manufacturer’s instructions. The amplified cDNA for each treatment were diluted five times with nuclease-free water before quantitative polymerase chain reaction (qPCR).

According to the miRNAs-TF network analysis, eight differentially expressed TFs were selected to validate their expression levels. Coding DNA sequences (CDS) obtained from WormBase ParaSite using the Biomart tool were used to design specific primers using PrimerQuest™ Tool (Integrated DNA Technologies, https://www.idtdna.com › pages › tools › primerquest). The details of each selected TF and the characteristics of each primer are summarized in **Additional file 1. Table S10**.

The qPCR reactions were performed using Rotor-Gene Q (Qiagen, Germany) with the following reaction mixture: 3 µL of diluted cDNA (1:5) as template, 0.5 µL of each primer (10 µM), 10 µL of SYBR Green qPCR Master Mix (Yekta Tajhiz Azma, Iran), and DNase free water in a final reaction volume of 20 µL. A non-template control (NTC) was included for each run. All qPCR reactions for each sample and each gene were performed in triplicate. The amplification conditions were as follows: initial activation 95 °C for 5 min, 40 cycles of 95 °C for 15 s, 60 °C for 30 s, and 72 °C for 30 s. Melt curve of each amplified product was analyzed to assess the amplification specificity for each gene following amplification cycles with ramping increments of 0.1 °C/s from 60 to 99 °C., the specificity of the amplicons was also analyzed by agarose gel electrophoresis. The LinRegPCR [57] software was used to calculate the PCR efficiency of all primer pairs and the NormFinder [58] was used to find the most stable reference genes for qPCR analysis. The comparative 2^−∆∆CT^ method was used to estimate relative gene expression levels, and the mean of fold changes was utilized to compare groups [59].

## Electronic supplementary material

Below is the link to the electronic supplementary material.

**Additional file 1: Table S1.** List of Echinococcus granulosus Transcription factors with differentially expressed genes in early stages of strobilar development. **Table S2.** List of predicted targets derived from miRanda, PITA, and RNA22 and their comparison with 818 differentially expresssed genes and 41 differentially expressed transcription factors. **Table S3.** The summary of gene enrichment data obtained from KEGG database for predicted targets of selected differentially expressed miRNAs. **Table S4.** Prediction of protein?protein interaction network for six differentially expressed TFs by using the Search Tool for Recurring Instances of Neighboring Genes (STRING). **Table S5.** Comparison of the in vivo, in vitro and the RNA-seq data of the selected differentially expressed TFs and their related miRNAs. **Table S6.** Calculation of the Betweenness, Degree and Closeness centrality of the predicted network by using the cytoHubba (version 0.1). **Table S7.** List of Echinococcus granulosus differentially expressed genes in early stages of strobilar development according to Debarba et. al 2020. **Table S8.** List of Echinococcus granulosus miRNAs with differentially expressed genes in early stages of strobilar development according to Bai et. al 2020. **Table S9.** List of 396 Echinococcus granulosus Transcription factors and related DNA-binding domains/transcription factors families according to AnimalTFDB 3.0 database (394 unique TFs). **Table S10.** Sequences of forward and reverse primers of eight differentially expressed transcription factors and references genes used in qPCR validation.

**Additional file 2:** The Gene Ontology enrichment of the clusters of related genes to each differentially expressed miRNA in early strobilar development of *Echinococcus granulosus* protoscoleces.

## Data Availability

The datasets analyzed during the current study are publicly available on GenBank under the accession number SRP131874 and Bai et al. article [5]. All generated or analyzed datasets are available from the corresponding author on reasonable request.

## Abbreviations

PEP: Pepsin- treated group
12 h: Pepsin-treated group after 12 h
24 h: Pepsin-treated group after 24 h
TFs: Transcription factors
DEGs: Differentially expressed genes
DETFs: TFs with differential expression
DEmiRNAs: Differentially expressed miRNAs.
